# Supplementary material for: CAF-Released Exosomal miR-20a-5p Facilitates HCC Progression via the LIMA1-Mediated β-Catenin Pathway
Source: Cells. 2022 Nov 30;11(23):3857. doi: 10.3390/cells11233857 (PMC9740131; doi:10.3390/cells11233857)
Supplement: Supplementary file 1 [file cells-11-03857-s001.zip › cells-2004068-supplementary.pdf]

Supporting Information

CAF-released exosomal miR-20a-5p facilitates HCC progression via the LIMA1-mediated β-catenin pathway

Yong Qi[1,2], Haibo Wang[1,2], Qikun Zhang[1,2], Zhiqiang Liu[2], Tianbing Wang[3], Zhengsheng Wu[4] *,Wenyong Wu[1,2,3] *

Table of contents:

Supplementary Figure S1

Supplementary Figure S2

Supplementary Figure S3

Supplementary Figure S4

Supplementary Figure S5

Supplementary Figure S6

Supplementary Figures and figure legends

Supplementary Figure S1

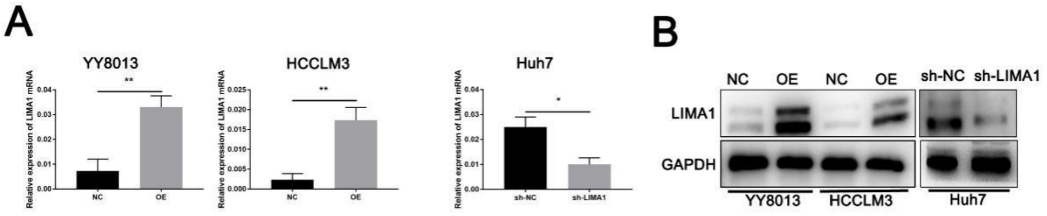

Supplementary Figure S1. LIMA1 was overexpressed or knocked down in HCC cell lines. LIMA1 overexpression in YY8103 and HCCLM3 cells and LIMA1 knockdown in Huh7 cells were determined by RT-qPCR (A), and western blotting (B). (NC, normal control; OE, LIMA1 overexpression).**P < 0.05, **P < 0.01, ***P < 0.001.*

Supplementary Figure S2

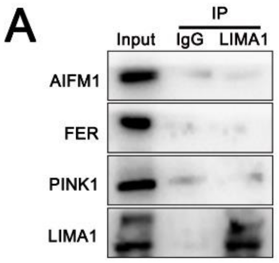

Supplementary Figure S2. LIMA1 could not bind to AIFM1, FER and PINK1. (A) IP assay was performed to detect the binding of LIMA1 to AIFM1, FER and PINK1.

Supplementary Figure S3

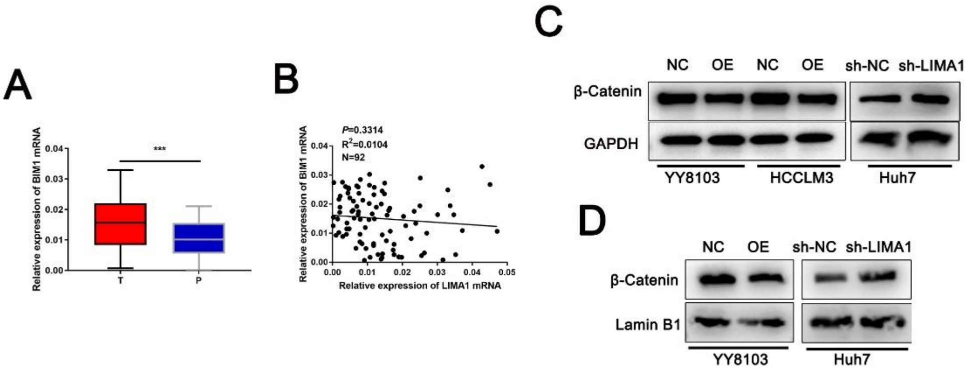

Supplementary Figure S3. BMI1 was upregulated in HCC and mediated the activation of wnt/β-catenin pathway. (A) The expression level of BMI1 mRNA and (B) the correlation of

LIMA1 and BMI1 were examined by qRT-PCR in ninety-two pairs of HCC patients. (C) β-catenin expression was detected with LIMA1 knockdown or overexpression. (D) Nuclear proteinswereobtainedtoexaminetheβ-catenin expression level with LIMA1 knockdown or overexpression. (NC, normal control; OE, LIMA1 overexpression). *$P < 0.05$, **$P < 0.01$, ***$P < 0.001$.*

Supplementary Figure S4

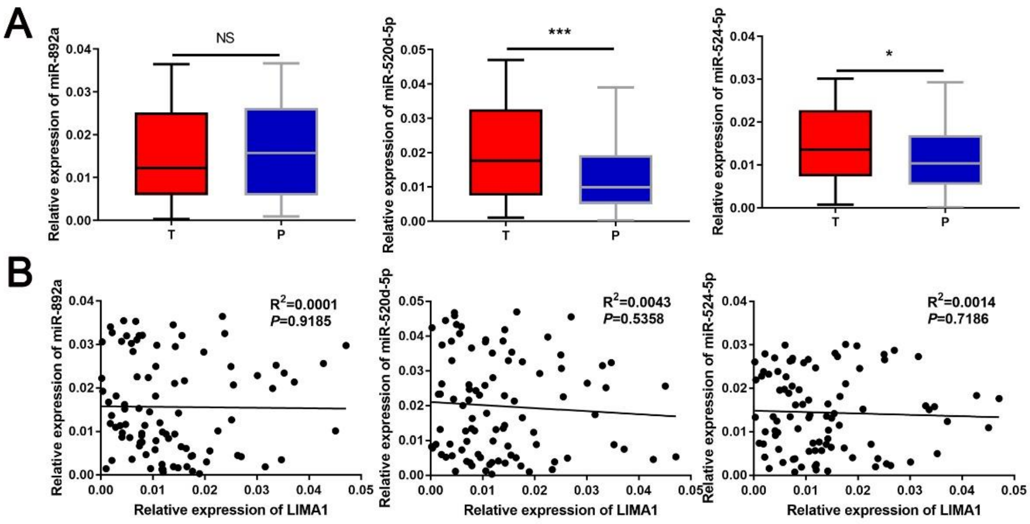

Supplementary Figure S4. Expression of miR-892a, miR-520d-5p and miR-524-5p and their correlation were analyzed in HCC tissues. (A) Expression of miR-892a, miR-520d-5p and miR-524-5p in HCC tissues was examined by RT-qPCR. (B) The correlation between miR-892a, miR-520d-5p, miR-524-5p and LIMA1 was analyzed in HCC tissues. *NS: $P > 0.05$, *$P < 0.05$, **$P < 0.01$, ***$P < 0.001$.*

Supplementary Figure S5

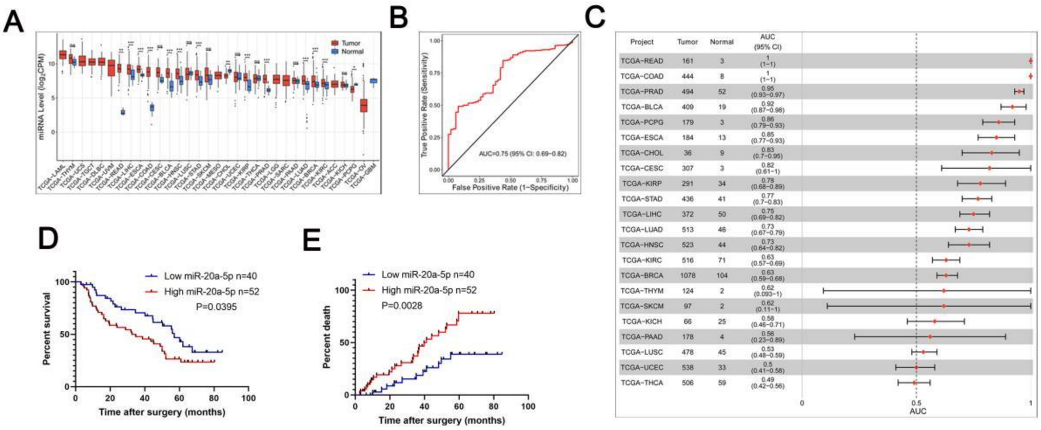

Supplementary Figure S5. Expression of miR-20a-5p in various cancers and its clinical significance were investigated based on TCGA database, and the survival curves of miR-20a-5p in the miR-20a-5p-high and miR-20a-5p-low groups. (A) The expression level of miR-20a-5p in multiple type of cancers was determined based on TCGA database. (B) and (C)

The AUC value of miR-20a-5p in HCC and many other cancers was determined based on TCGA database. (D) Overall survival (OS) and (E) recurrence-free survival (RFS) of HCC patients in the miR-20a-5p-high and miR-20a-5p-low groups.

Supplementary Figure S6

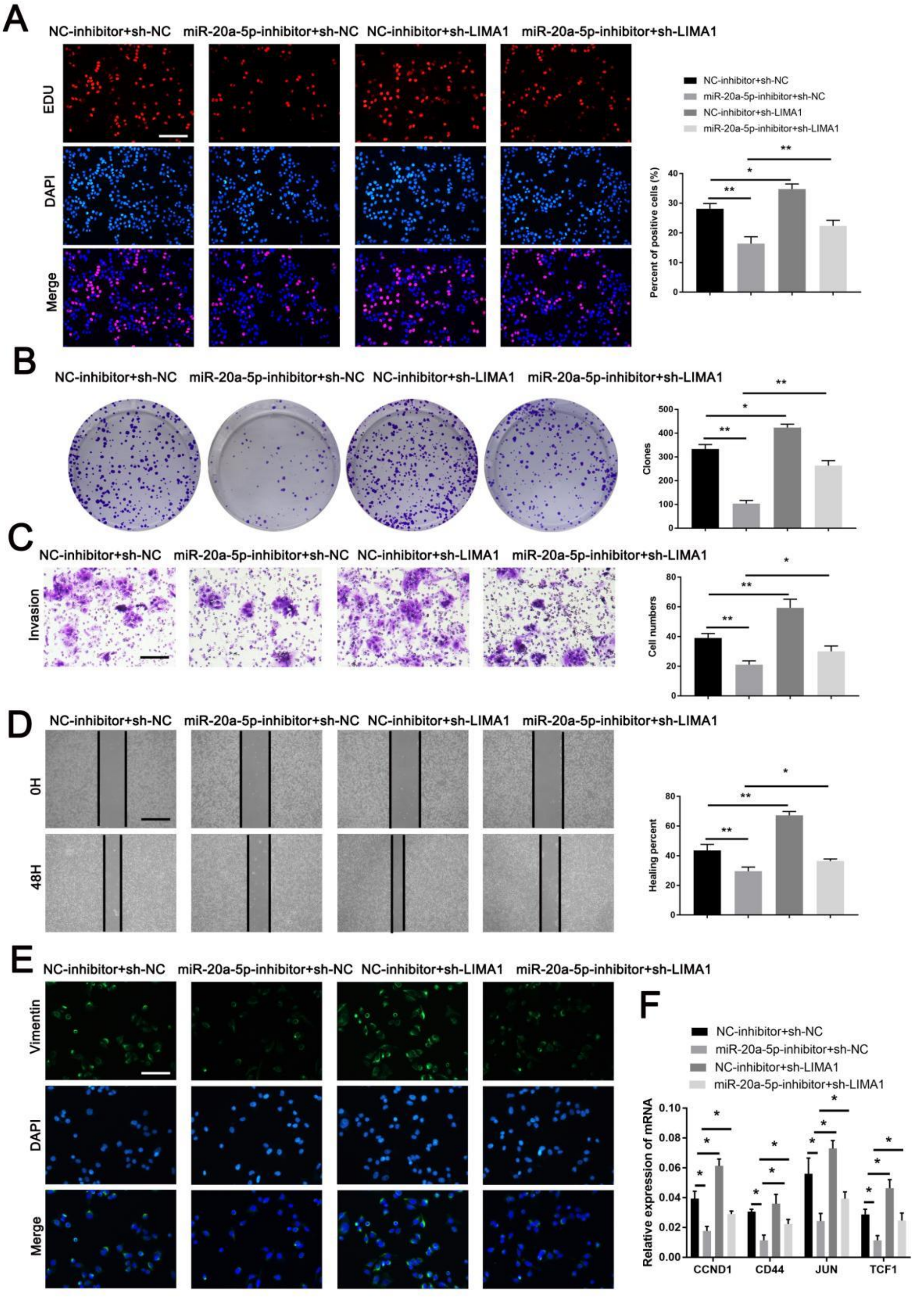

Supplementary Figure S6. LIMA1 knockdown restored the oncogenic role of miR-20a-5p. (A) Colony formation, (B) EdU, (C) Transwell, (D) wound healing assays and (E) immunofluorescence for vimentin were performed to detect the rescued effects of LIMA1 on miR-20a-5p in YY8103 cells cotransfected with NC-inhibitor+sh-NC,

miR-20a-5p-inhibitor+sh-NC, NC-inhibitor+sh-LIMA1 and miR-20a-5p-inhibitor+si-LIMA1.
(F) The downstream molecules of β-catenin were examined by RT-qPCR in YY8103 cells cotransfected with NC-inhibitor+sh-NC, miR-20a-5p-inhibitor+sh-NC, NC-inhibitor+sh-LIMA1 and miR-20a-5p-inhibitor+sh-LIMA1 to determine the rescued effects of LIMA1 on miR-20a-5p. *$*P < 0.05$, $**P < 0.01$, $***P < 0.001$.*
